# Incidence and causes of major amputation in patients with diabetic foot ulcers: data from a retrospective study

**DOI:** 10.1007/s00592-025-02577-1

**Published:** 2025-09-04

**Authors:** Marco Meloni, Luigi Uccioli, Aikaterini Andreadi, Laura Giurato, Valeria Ruotolo, Maria Romano, Alessandro Minasi, Ermanno Bellizzi, Federico Rolando Bonanni, Martina Salvi, Alfonso Bellia, Davide Lauro

**Affiliations:** 1 Department of Systems of Medicine, University of Tor Vergata, Rome, 00133 Italy; 2 Division of Endocrinology and Diabetology, Department of Medical Sciences, University Hospital Fondazione Policlinico Tor Vergata, Rome, Italy; 3 Department of Endocrinology and Diabetology, CTO Hospital, Rome, 00145 Italy; 4 Division of Endocrinology and Diabetology, Department of Medical Sciences, Department of Systems of Medicine, University Hospital Fondazione Policlinico Tor Vergata, University of Tor Vergata, Viale Oxford 81, Rome, 00133 Italy

**Keywords:** Diabetes, Diabetic foot, Amputation, Peripheral arterial disease

## Abstract

**Aim:**

The study aimed to evaluate the rate and causes of major amputation in patients with diabetic foot syndrome.

**Methods:**

The current study is a retrospective observational study including consecutive patients referred to a tertiary-level diabetic foot service from January 2020 to November 2023 due to a new diabetic foot problem requiring hospital admission. All patients had been managed by a multi-disciplinary diabetic foot team (MDFT) through a pre-set limb salvage protocol including the management of peripheral arterial disease, infection, foot offloading, and comorbidities. At 1 year of follow-up, the following outcomes measures were evaluated: rate of major amputation, clinical characteristics of amputees, and causes of major amputation.

**Results:**

Overall, 1226 patients referring for a diabetic foot problem and requiring hospitalization were screened for the study. Among them, 30 (2.4%) patients experienced major amputation. Amputees had 69.9±10.7 years, the majority were male (73.3%) with a prevalence of type 2 diabetes (93.3%) and a long diabetes duration (25.2±9.8 years). They showed several comorbidities such as ischaemic heart disease (83.3%), heart failure (46.7%), end-stage-renal-disease (26.7%), and in addition high rate of peripheral arterial disease (PAD) (86.7%), infected wounds (98.3%), and osteomyelitis (90%). Major amputation was mainly related to untreatable limb ischemia (failure of revascularization procedure) in 56.7% of cases, calcaneus osteomyelitis and necrotizing fasciitis in 16.7% of cases, and tarsal osteomyelitis in 10% of cases.

**Conclusions:**

The rate of major amputation was very low in this population managed by a MDFT. PAD was the main cause of major amputation.

**Supplementary Information:**

The online version contains supplementary material available at 10.1007/s00592-025-02577-1.

## Introduction

Diabetic foot disease (DFD) is a clinical, social, and economic burden. Recent data have estimated that the prevalence of DFD affects approximately 199 million people, involving 2.6% of the global population [1]. Among persons with diabetes, 19–34% can develop a foot ulcer throughout their life [2].

DFD also represents a significant cause of death, with a 5-year mortality incidence of 33–60% in patients with a history of diabetic foot ulcer (DFU) [3]. The risk of death at 5 years for patients with DFUs is 2.5 times higher than patients with diabetes without a foot ulcer [3]. Major amputation is an independent risk factor for mortality, and the risk of mortality is dramatically increased in patients undergoing major amputation [4, 5], being higher than 70% at 5 years [3, 5].

Data on significant amputation in persons affected by diabetes, with or without DFU, are not so easy to be detected, being studies on DFD very different with not homogeneous populations, different inclusion criteria and follow-up.

In a study on 21 countries coming from the geographic area of the Organization for Economic Cooperation and Development (OECD) [6], conducted between 2000 and 2013, Carinci et al. found a global mean reduction in major amputations from 182.9 to 128.3 cases per 100,000 subjects with diabetes (− 30.6%) during the observation period.

Despite the decreasing trend, the amputation rate resulted significantly higher when compared to the incidence of major amputation in the general population (from 10.8 to 7.5 per 100,000 subjects), confirming the contribution of diabetes as an independent risk factor for non-traumatic lower limb amputation [1, 6, 7].

Lower rates of major amputation have been reported in some countries such as Italy (49.4 per 100,000 subjects) [6], where diabetic foot care is managed for a long time in dedicated diabetic foot services through a multidisciplinary team, which has been reported to be essential for improving outcomes such as wound healing and limb salvage [8].

Reducing lower extremities amputations is the main clinical objective for health care professionals (HCPs) managing DFD, and limb salvage is considered the primary marker of the quality of care. The reduction of avoidable major amputations in persons with diabetes is a daily challenge for healthcare providers and healthcare systems.

Nonetheless, data on major amputation, such as incidence and causes, are not usually homogeneous and can vary according to different settings, populations, and management. Based on this background, authors aimed to evaluate the incidence and causes of major amputation in a population with DFUs managed in a dedicated tertiary-level diabetic foot service.

Authors aimed to collect epidemiological data and highlighting the current causes of failure for improving next generation treatments.

## Materials and methods

**Fig. 1:**
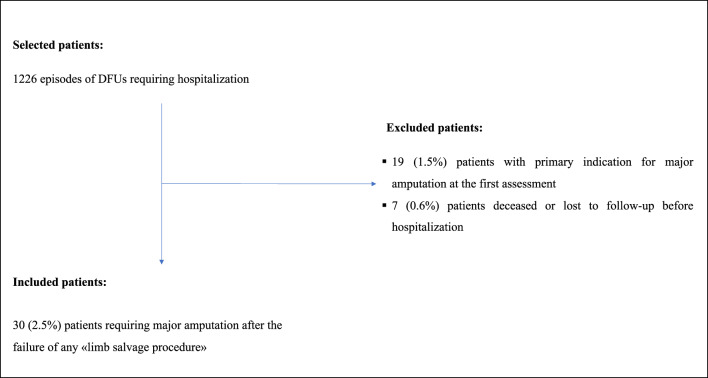
Flow chart of included patients

The current study is a retrospective observational study. Consecutive patients referring to a tertiary-level diabetic foot service (DiFoSe) from January 2020 to November 2023 due to a new diabetic foot problem were considered for the current study. Patients who presented at the first assessment an extensive foot tissue loss not suitable for any procedure of limb salvage were excluded and referred to general surgeons (Fig. 1). The included patients were managed in the Endocrinology and Diabetes Unit, a tertiary level DiFoSe, at the University Hospital “Fondazione Policlinico” Tor Vergata, Rome, Italy.

All patients have been managed by a multidisciplinary diabetic foot team (MDFT) through a pre-set limb salvage protocol in accordance with the International Working Group on the Diabetic Foot (IWGDF) guidelines, including lower limb revascularization in the case of ischaemic/neuro-ischaemic DFU, antibiotic therapy (and surgery if required) in the case of infection, offloading of affected foot, surgery according to each specific clinical case [9]. In addition, metabolic dysfunction, diabetic complications, and concomitant co-morbidities were closely managed.

The MDFT included diabetologists, endocrinologists, interventional radiologists, vascular surgeons, podiatrists, and nurses. Diabetologists were the case managers, overseeing all medical aspects, including glycaemic and lipid control, cardiovascular and renal assessment, foot infection, and nutritional support. Among diabetologists, those skilled in diabetic foot surgery were dedicated to surgical wound procedures below-the-ankle.

At the assessment demographic and clinical data were recorded, such as wound characteristics.

### Clinical features

Hypertension was considered in the case of current antihypertensive therapy; hypercholesterolemia was considered in the case of statin therapy or in the case of impaired low-density-lipoproteins (LDL) (> 55 mg/dl) at the assessment requiring statin therapy [10]. The presence of ischaemic heart disease (IHD) was defined in the case of previous acute coronary syndrome or coronary revascularization, evidence of angina, significant changes on electrocardiography (ST-elevation or depression, q wave, inversion of the T wave, new left bundle branch block). Heart failure (HF) was considered in the case of typical symptoms and echocardiographic signs of HF: reduced left ventricular ejection fraction (LVEF) (< 40%) or normal or only mildly reduced LVEF and elevated levels of brain natriuretic peptides (BNP > 35 pg/ml and/or NT-proBNP > 125 pg/ml) with not dilated left ventricle (LV) associated to relevant structural heart disease (LV hypertrophy/left atrial enlargement) and/or diastolic dysfunction [11]. Patients were considered smokers only in case of smoking habit at the time of assessment. Cerebrovascular disease was considered in the case of previous cerebral ischemia, previous carotid artery revascularization, and a new diagnosis of atherosclerotic plaque occluding carotid artery more than 70% suitable for revascularization. The rate of patients on end-stage-renal-disease (ESRD) requiring dialysis was reported.

### Wound assessment

Wound features were recorded at the first assessment according to IWGDF definitions [12]. Infected DFUs were defined according to at least two clinical signs (redness, warmth, swelling, induration, tenderness, pain, purulent secretion) [9]. Osteomyelitis was considered for deep ulcer involving the bone, confirmed by radiological evaluation (or magnetic resonance) and positive microbiological analysis [9].

Peripheral neuropathy was defined in the case of loss of peripheral sensitivity detected through vibration perception (128 Hz tuning fork) and/or Semmes-Weinstein 10-g monofilament [9].

Neuro-ischaemic/ischaemic DFU included in the current study were considered in the case of either no palpable distal pedal pulses, ankle-brachial index (ABI) < 0.5 and/or transcutaneous oxygen pressure (TcPO2) < 30 mmHg requiring lower limb revascularization [9]. Bedside tests used for the vascular assessment were performed only by expert professionals working in this specific clinical setting (interventional radiologists/vascular surgeons) to avoid any risk of inter-observer variability.

All patients underwent a morphological evaluation of the vascular tree to identify arterial stenosis or occlusions using ultrasound duplex. Computed tomography or MRI were conducted only when the ultrasound duplex results were unclear, and further assessment was necessary to ascertain the severity of vascular plaques or the type of vascular intervention needed. The primary goal of revascularization procedures was to restore patency in all occluded arteries or, if not feasible, to focus on revascularizing the specific artery related to the wound.

### Outcome measures

The outcome measures were to evaluate the major amputation rate after one-year follow-up in patients requiring hospitalization for DFUs, major amputation causes, and the clinical characteristics of amputees. Major amputation was considered any amputation above-the-ankle.

The causes of major amputation were defined according to the reason for hospitalization at the assessment and for its treatment failure: Chronic limb threatening ischaemia (CLTI) was considered the cause of major amputation in the case of revascularization failure characterized by the absence of blood flow below-the-ankle or the lack of direct flow to the wound angiosome area, determining a progression of tissue loss and/or the persistence of foot/leg pain not allowing a conservative therapy; infection, including both soft tissue and/or osteomyelitis, was considered the cause of major amputation in the case of medical and surgical infection treatment failure, determining an extension of infected tissue not allowing a conservative/curative strategy due to the involvement of essential foot structures. In the specific case of osteomyelitis, the reason for major amputation was considered the primitive site of infection, regardless of the subsequent involvement of other bones (i.e., if the patient was admitted for calcaneus osteomyelitis, the same site was considered the cause of major amputation even though the infection had spread in the nearby soft tissues or bones).

The research was conducted in accordance with the principles embodied in the Declaration of Helsinki and local statutory requirements. The current study did not require Ethical approval according to the local policy.

All patients provided their verbal consent for data recording and being considered for the study.

### Statistical analysis

Statistical analysis was performed using SAS software (JMP12; SAS Institute, Cary, NC). Data were expressed as means ± SD and the continuous data for the outcome as a percentage.

## Results

Overall, 1226 patients referring for a DFU and requiring hospitalization were considered for the current study. Among the whole population, 19 (1.5%) were excluded due to primary indication for major amputation and 7 (0.6%) were deceased or lost to follow-up (see Fig. 1).

Overall, 30/1200 (2.5%) patients experienced major amputation after failing all procedures of limb salvage (Table 1).

**Table 1:** Baseline clinical and wound characteristics

Variable	Value (*n* = 30)
Sex (male) n (%)	22 (73.3%)
Age (years)	69.9±10.7
Diabetes Type (2) n (%)	28 (93.3)
Diabetes duration (years)	25.2±9.8
HbA1c mmol (%)	63±8 (7.9±2.9)
IHD n (%)	25 (83.3)
Heart failure	14 (46.7)
Hypertension n (%)	26 (86.7)
Dyslipidaemia n (%)	20 (66.7)
ESRD n (%)	8 (26.7)
PAD n (%)	26 (86.7)
Infection n (%)	28 (98.3)
Severe infection n (%)	22 (73.3)
Bone involvement n (%)	27 (90)
Ulcers size (> 5cm^2^) n (%)	28 (98.3)
Gangrene n (%)	24 (80)
Heel involvement n (%)	9 (30)
Ulcer recurrence n (%)	19 (63.3)
Minor amputation n (%)	18 (60)

The mean age of included patients was nearly 70 years; the majority were male with a prevalence of type 2 diabetes and a long diabetes duration (approximately 25 years) (Table 1).

From a clinical point of view, they showed several co-morbidities and a high incidence of cardiovascular risk factors, at the same time, they presented a severe involvement of the affected foot: the majority had an ischaemic DFU and nearly 100% had an infected DFU, more than 70% had a severe infection, in addition the largest part showed bone involvement (Table 1).

More than half of the patients received major amputation due to untreatable limb ischemia with a mechanical revascularization failure, followed by the presence of calcaneal osteomyelitis and necrotizing fasciitis at the first assessment (Table 2).

**Table 2:** Causes of major amputation

Variable	Values (*N* = 30)
Untreatable CLTI (revascularization failure) n (%)	17 (56.7)
Calcaneus osteomyelitis n (%)	5 (16.7)
Necrotizing fasciitis n (%)	5 (16.7)
Tarsal osteomyelitis n (%)	3 (10)

Three patients (10%) died after major amputation during their hospitalization.

## Discussion

The first remarkable data emerging from the current study is the low incidence of major amputation (2.5%) in a population of patients with DFU managed by an MDFT in a tertiary level diabetic foot service. The low rate of major amputation, carefully considering all potential bias of a retrospective analysis, showed a significant reduction on the trend of major amputation when compared to a similar population managed in our specific region.

Specifically, Uccioli et al. reported a major amputation incidence of 14.9% in patients with ischaemic DFUs managed between 2002 and 2007 and followed for a mean of 20 months [13], while Meloni et al. reported a 4.6% rate of major amputation in a population of outpatients and inpatients, including both neuropathic and ischaemic DFUs, managed between 2010 and 2018 and followed for at least 1 year [14].

Even though authors are not analytically documenting the reasons of this trend on major amputation reduction, some potential factors could be considered: the increase of early referral in patients with DFU by the reinforced network between hospital and community [15], the improved techniques in lower limb revascularization [16–19], the common use of autologous cell therapy for treating patients with no-option CLTI [20, 21], and the further implementation of the multi-disciplinary team approach [22].

Data on major amputation are very encouraging when compared to similar studies.

Faglia et al. reported a major amputations rate of 13.4% in patients with ischaemic DFUs followed for a mean of 6 years, with a rate of early major amputation of approximately 4.2% [23]. The rate of major amputation was significantly higher in those patients who did not receive peripheral revascularization or had a revascularization failure (59.2%) [23].

Recently, Fang et al. documented a 14.5% rate of major amputation at 5 years of follow-up in a population with diabetic foot disease, with most events occurring in the first year [24]. Kang et al. reported 17.6% of major amputation in a cohort of patients with ischaemic DFUs and CLTI managed by peripheral endovascular revascularization [25]. Gong et al. found a major amputation rate of 1.7% in a population hospitalized for DFUs between 2012 and 2020 [26], resulting more or less similar to that we found in our analysis (2.5%).

After identifying the major amputation incidence in patients with DFUs requiring major amputation, authors aimed to better describe the clinical pattern of amputees, including general health status, cardio-vascular profile, and wound characteristics at the first assessment.

The majority of patients were male (approximately 73%) as usually appear for complicated DFUs [14, 27], they showed a long history of diabetes (a mean of 25 years) and a poor metabolic control (nearly 8% of HbA1c). The duration of diabetes seems longer than that usually recorded for patients admitted for DFUs (nearly 20 years) [22], and long diabetes duration such as poor glycemic control, are already reported as two risk factors for major amputation [26].

The very interesting data from a clinical point of view is the severe cardiovascular profile of amputees: 83.3% had IHD, 46.7% had a history of heart failure, and 26.7% were under dialysis treatment. This framework should be considered not surprising, being heart and renal complications two well-known predictors of major amputation and mortality in patients with DFUs, mainly ischaemic DFUs, as already documented in some previous studies [14, 23, 28]. Nonetheless, the incidence of heart complications, including both IHD and heart failure, appears extremely high when compared to some previous studies on patients affected by DFUs, where usually the prevalence of IHD and heart failure was estimated to be < 50% and 30%, respectively [14, 23, 27].

If amputees showed a very severe cardiovascular profile, the same severity was reported for that regarding the DFUs aspects: most wounds were ischaemic, nearly the totality was infected and deep to the bone, and 30% of theme involved the rearfoot. All the mentioned characteristics are related to worse outcomes, including non-healing, minor, and major amputation [14, 27]. The most impacting data from a clinical point of view is that 63.3% of amputees presented a history of previous ulceration and 60% a history of minor amputation. The prevalence of previous ulceration/minor amputation among amputees is extremely high, documenting that ulcer recurrence is a real burden and challenge in patients with DFD, and reinforcing the concept that readmission for ulcer recurrence increases the risk of worse outcomes [29].

Regarding the leading causes of major amputation, CLTI, and specifically revascularization failure, resulted the main reason in more than half of patients. This data confirms that revascularization failure, defined as no-option CLTI, is the most significant risk factor for amputation, as reported in different studies [13, 14, 23, 30]. A recent retrospective study analyzing the incidence and risk factors for amputation confirms that peripheral arterial disease increases 10 times the risk of major amputation in patients with diabetes and foot ulcers [31]. The absence of adequate blood flow does not allow for adequate reconstructive surgery and, in some circumstances, could promote the progression of the infectious process.

The other causes of major amputation were related to the presence of severe infected DFU, in the majority of cases involving the bone. The current study showed that calcaneus osteomyelitis and necrotizing fasciitis are the main causes of major amputation after untreatable CLTI. Heel ulcers, specifically calcaneus osteomyelitis, are very difficult to be managed and they have been recognized as independent risk factor for non-healing and amputation [32–35]. This could be related to both anatomical and biological reasons, being ulcers located in the rearfoot very hard to be treated by first intention and having the heel area a poor blood perfusion due to the absence of muscle tissues [35]. In addition, in the case of osteomyelitis, the complete removal of calcaneus is not feasible due to biomechanical factors. Necrotizing fasciitis is a severe soft tissue infection that usually quickly spreads into the different foot compartments; in several cases, mainly in those with concomitant CLTI, necrotizing fasciitis shows a poor response to surgery and antibiotic therapy. Necrotizing fasciitis is also one of the main causes of late referral when the infection is not managed in the initial phase after its onset. This data could confirm that late referral remains a direct or indirect reason for amputation in patients with DFUs, mainly in those with infected and/or ischaemic wounds [15].

The current study showed a very low rate of major amputation in a population of patients with DFUs when compared to the same data collected many years before in the same working setting and when compared to literature data. Many factors could influence the high rate of limb salvage, including the reduction of late referral, the implementation of the multi-disciplinary team, improvements in vascular interventions, and new technologies.

The study has some limitations. It is a monocentric study and retrospective observational study. The follow-up was 1 year, and longer follow-up could increase the chance of potential major amputation. Some variables, including both social and economic factors, that can interfere with outcomes are not considered. Patients with primary indication to major amputation are not included in the study, and causes of amputation are not taken into consideration, having these patients a severe tissue loss not suitable for limb reconstruction.

Nonetheless, patients included in the study have been treated by the same multi-disciplinary team and received similar limb preservation procedures, promoting a homogeneous population in terms of inclusion criteria and treatment.

## Conclusions

The multi-disciplinary team approach can contain the risk of major amputation in patients with DFUs. Untreatable CLTI seems to be a major risk factor for amputation. New strategies for patients with no-option CLTI should be implemented in the next generation treatments.

## Supplementary Information

Below is the link to the electronic supplementary material.

Supplementary Material 1
