# Supplementary material for: Incidence and causes of major amputation in patients with diabetic foot ulcers: data from a retrospective study
Source: Acta Diabetol. 2025 Sep 4;63(1):97–103. doi: 10.1007/s00592-025-02577-1 (PMC12847106; doi:10.1007/s00592-025-02577-1)
Supplement: Supplementary file 1 — Supplementary Material 1 [file 592_2025_2577_MOESM1_ESM.pdf]

The authors have no conflicts of interest to declare that are relevant to the content of this article.
